# Protein body formation in stable transgenic tobacco expressing elastin-like polypeptide and hydrophobin fusion proteins

**DOI:** 10.1186/1472-6750-13-40

**Published:** 2013-05-10

**Authors:** Sonia P Gutiérrez, Reza Saberianfar, Susanne E Kohalmi, Rima Menassa

**Affiliations:** 1 Department of Biology, University of Western Ontario, London, ON, Canada; 2 Southern Crop Protection and Food Research Centre, Agriculture and Agri-Food Canada, London, ON, Canada

**Keywords:** Protein body, Protein body formation, Elastin-like polypeptide, ELP, Hydrophobin I, HFBI, Tobacco, Transgenic expression, Molecular farming, Green fluorescent protein

## Abstract

**Background:**

Plants are recognized as an efficient and inexpensive system to produce valuable recombinant proteins. Two different strategies have been commonly used for the expression of recombinant proteins in plants: transient expression mediated by *Agrobacterium*; or stable transformation of the plant genome. However, the use of plants as bioreactors still faces two main limitations: low accumulation levels of some recombinant proteins and lack of efficient purification methods. Elastin-like polypeptide (ELP), hydrophobin I (HFBI) and Zera® are three fusion partners found to increase the accumulation levels of recombinant proteins and induce the formation of protein bodies (PBs) in leaves when targeted to the endoplasmic reticulum (ER) in transient expression assays. In this study the effects of ELP and HFBI fusion tags on recombinant protein accumulation levels and PB formation was examined in stable transgenic *Nicotiana tabacum*.

**Results:**

The accumulation of recombinant protein and PB formation was evaluated in two cultivars of *Nicotiana tabacum* transformed with green fluorescent protein (GFP) fused to ELP or HFBI, both targeted and retrieved to the ER. The ELP and HFBI tags increased the accumulation of the recombinant protein and induced the formation of PBs in leaves of stable transgenic plants from both cultivars. Furthermore, these tags induced the formation of PBs in a concentration-dependent manner, where a specific level of recombinant protein accumulation was required for PBs to appear. Moreover, agro-infiltration of plants accumulating low levels of recombinant protein with p19, a suppressor of post-transcriptional gene silencing (PTGS), increased accumulation levels in four independent transgenic lines, suggesting that PTGS might have caused the low accumulation levels in these plants.

**Conclusion:**

The use of ELP and HFBI tags as fusion partners in stable transgenic plants of tobacco is feasible and promising. In a constitutive environment, these tags increase the accumulation levels of the recombinant protein and induce the formation of PBs regardless of the cultivar used. However, a specific level of recombinant protein accumulation needs to be reached for PBs to form.

## Background

The fast-growing demand of recombinant proteins and the limitations of traditional production systems (bacteria, fungi and mammalian cell cultures) have outlined the need for alternative strategies of high-level recombinant protein production [1-3]. Production of recombinant proteins in plants has been used for proof of concept production of a wide range of proteins including industrial enzymes and biopharmaceuticals [4,5]. The first plant-made pharmaceutical for human use was recently approved by the US Food and Drug Administration [6].

A wide range of host plants have been developed and tested for molecular farming, however tobacco still remains one of the favorite hosts for the commercial production of recombinant proteins [7,8]. Genetic manipulation of tobacco is well established, and its high biomass yield (more than 100,000 kg of tissue per hectare) facilitates large-scale production of recombinant proteins [2,3]. Moreover, different *Nicotiana* varieties have been characterized according to their agronomic properties and ability to accumulate recombinant proteins, therefore facilitating the search for the most effective tobacco host for recombinant protein production [9]. Since the tobacco expression platform is based on leaves which are harvested before flowering, the risk of pollen or seed dispersal of the transgene is reduced [10]. Furthermore, tobacco is a non-food, non-feed crop, which lowers the risk of plant-made recombinant proteins entering the human and animal food chain [3,11]. Although tobacco is inherently biosafe, the commercial viability of molecular farming with this plant species has been limited by two main factors: low accumulation levels of some recombinant proteins and lack of efficient and scalable protein purification methods [8,9].

A wide variety of strategies have been tested in plant-based systems to increase the stability and yield of recombinant proteins. In recent years, fusion protein technology has been used to enhance recombinant protein accumulation in heterologous systems. Among these, oil body-targeted oleosin fusion proteins and ER-targeted fusion proteins with elastin-like Polypeptide (ELP), hydrophobin I (HFBI) and Zera® have been of particular interest as they improve accumulation and stability of recombinant proteins in plants, and assist in the subsequent purification process of the recombinant proteins [12-18].

ELPs are synthetic polypeptides made up of a repeating five amino acid motif (Val-Pro-Gly-Xaa-Gly) similar to repetitive pentapeptides of the mammalian protein elastin. The guest amino acid (Xaa) can be any amino acid except proline. Upon an increase in temperature, soluble ELPs undergo a reversible transition into β spiral structures resulting in hydrophobic, insoluble aggregates. The transition temperature (T_t_) at which phase transition occurs depends on the number of pentapeptide repeats and on the guest amino acid [19,20]. This property of ELPs can be transferred to their fused protein partner and facilitates the purification of target proteins with a rapid, non-chromatographic purification method known as inverse transition cycling (ITC) [21].

Protein fusions with synthetic ELP tags retrieved to the ER using a C-terminal peptide (H/KDEL) have been successfully produced in plants and purified with ITC. Some examples include fusions with cytokines, antibodies and spider silk, all produced in transgenic tobacco plants, reviewed by Floss *et al.*[22].

Hydrophobins are a group of small, surface-active proteins originally identified in filamentous fungi [23]. These proteins play an important role in fungal growth and development involving adaptation of fungi to their environment [24,25]. One remarkable feature of these proteins is that one part of their surface is made of hydrophobic aliphatic side chains that form an exposed hydrophobic patch on one side of the protein [25]. Alternatively to a core stabilized by hydrophobic interactions, hydrophobins possess a characteristic pattern of eight conserved cysteine (Cys) residues, which form four intramolecular disulfide bridges that convey a high degree of protein stability [24]. Due to their structural properties, hydrophobins can self-assemble into an amphipathic protein membrane at hydrophilic-hydrophobic interfaces [26,27]. Therefore, purification of HFBI can be facilitated by using a two-step surfactant-based aqueous two-phase system (ATPS) [28]. Importantly, when fused to other proteins, hydrophobins can alter the hydrophobicity of the fusion partner allowing for simple, rapid, efficient, scalable, and inexpensive purification using ATPS [29].

Fusions with hydrophobin I (HFBI) from *Trichoderma reesei*[23] have been successfully used to overexpress and purify recombinant proteins from *Trichoderma* sp., insect cells and plant tissues [29-31]. Transient expression of endoplasmic reticulum (ER) targeted green fluorescent protein (GFP) fused to ELP and HFBI tags has shown that ELP and HFBI not only increase accumulation levels of the fused recombinant protein, but also induce the formation of ER-derived protein bodies (PBs) in leaves of *Nicotiana benthamiana*[14,30]. These PBs are comparable to the ones observed in leaves of stably or transiently transformed tobacco plants with Zera® or zeolin fusion proteins [15,32-34]. It is thought that these induced PBs enhance the accumulation levels of fusion proteins by stably storing large amounts of recombinant proteins without affecting the normal growth and development of the host plants [8,15,30].

Analysis of ELP and HFBI fusions for protein recovery and biological activity showed that the proteins of interest maintain their functionality which further confirmed the potential of the fusion protein approach [13,22,30]. However, the majority of successful examples in which massive amounts of fused proteins were produced and PBs were induced have been carried out in transient expression systems using *Agrobacterium* infiltration. In this study, we evaluated the effect of ER-targeted GFP-ELP and GFP-HFBI fusions in stable transgenic tobacco plants. Our results demonstrate that both of these tags increase accumulation levels of GFP and induce the formation of PBs in a concentration-dependent manner where a threshold level of accumulation is necessary for PB formation.

## Results

### Generation of transgenic tobacco (*N. tabacum*) plants

**Figure 1:**
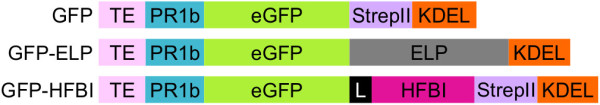
**Schematic representation of constructs used to generate transgenic tobacco plants.** TE, Translational enhancer from the tobacco tCUP promoter. PR1b, Secretory signal peptide from the pathogenesis related protein 1. eGFP, Enhanced green fluorescent protein. L, Linker (GGGS)_3_. HFBI, Hydrophobin I tag. ELP, Elastin-like polypeptide tag. StrepII, Fusion tag for detection and purification of recombinant protein. KDEL, ER retention peptide. Size of each element of the DNA constructs is not proportional to the actual sequence length [14,30].

To evaluate the effect of ELP and HFBI tags on accumulation levels of GFP and their potential ability to induce PB formation in transgenic tobacco, previously published plant expression vectors (GFP, GFP-ELP and GFP-HFBI) [14,30] were used for tobacco stable transformation (Table 1). These constructs were designed to express ER-targeted GFP, GFP-HFBI and GFP-ELP fusion proteins in tobacco plants under the control of the double enhanced cauliflower mosaic virus (CaMV) 35S promoter [35], a tCUP translational enhancer [36], and the nopaline synthase (nos) terminator [37] in the plant expression vector pCaMterX [38] (Figure 1), and were reported to increase expression levels of GFP and also to induce formation of PBs when transiently expressed in *N. benthamiana* leaves [8,30]. As well, two *Nicotiana tabacum* cultivars, I64 and 81V9, were shown to be among the most effective candidates for production of recombinant proteins in tobacco with respect to several properties including growth rate, leaf biomass yield, high soluble protein levels and low alkaloid content [9]. Although cv. I64 produces higher biomass than cv. 81V9, it has higher alkaloid levels, and elevated alkaloid levels may limit the therapeutic applications of whole plant tissue. Therefore, depending on the end use of the protein of interest (therapeutic vs. industrial; purified vs. whole tissue administration), one cultivar may be more desirable than the other. Therefore, we generated twenty four independent transgenic I64 lines with each of the three constructs to conduct a construct comparison, and twenty four transgenic 81V9 lines expressing the GFP-HFBI construct were produced to allow for a comparison of the two cultivars. All transgenic plants displayed a normal phenotype when compared with untransformed plants.

**Table 1:** Tobacco transgenic lines produced with the three expression constructs

***N. tabacum *cultivar**	**Construct name**	**Recombinant protein**	**Number of transgenic lines**
I64	GFP	GFP, targeted to the ER	24
I64	GFP-ELP	GFP with ELP tag, targeted to the ER	24
I64	GFP-HFBI	GFP with HFBI tag, targeted to the ER	24
81V9	GFP-HFBI	GFP with HFBI tag, targeted to the ER	24

### Quantification of recombinant proteins in transgenic tobacco lines

**Figure 2:**
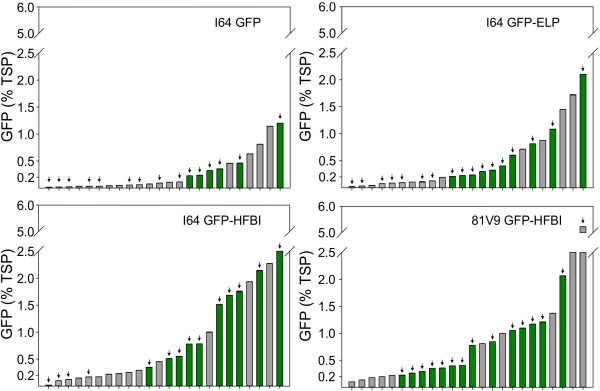
**Distribution of GFP, GFP-ELP and GFP-HFBI accumulation in independent transgenic tobacco lines.** Dark green bars, plants with PBs. Gray bars, transgenic lines which did not display PB formation or which were not analyzed by confocal microscopy. Arrows identify transgenic plants analyzed by confocal microscopy. The X-axis represents independent transgenic T_0_ lines, and the Y-axis represents the amount of GFP produced by each transgenic T_0_ line as determined by dot-blot analysis of triplicate loading of each sample.

GFP accumulation levels were quantified for tobacco plants expressing GFP, GFP-ELP and GFP-HFBI. The observed distribution of recombinant protein accumulation in different stable transgenic lines reflected the typical trend of a transgenic population where variability in accumulation levels across the primary transgenic lines is expected (Figure 2). This variability can be explained by positional effect of the transgene insertion within the genome, transgene copy number and/or silencing mechanisms suppressing transgene expression [39,40].

In general, most unfused GFP-expressing plants had lower accumulation of GFP than plants expressing GFP-ELP and GFP-HFBI; accumulation levels appeared to be higher in GFP-HFBI lines than in GFP-ELP lines, and no significant differences were detected between 81V9 and I64 populations expressing GFP-HFBI (Figure 2).

**Figure 3:**
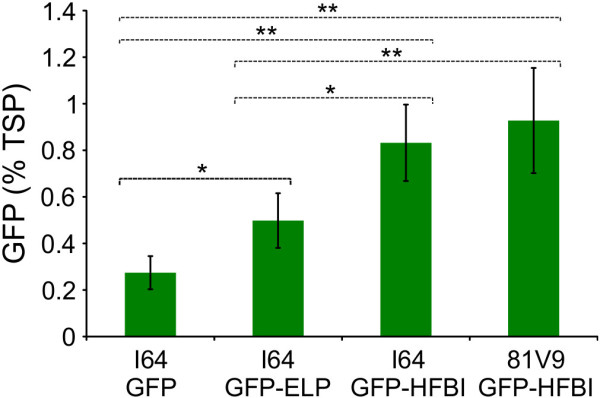
**Comparison of GFP accumulation in four transgenic tobacco groups.** Each column represents the mean value of 24 independent transgenic plants regenerated for each transformed construct and error bars indicate the standard error of the mean. Dotted lines indicate pairwise significant differences at *P < 0.1; **P < 0.05 using the Wilcoxon-Mann–Whitney test between the two groups they delineate (e.g. the top dotted line represents a comparison of I64 GFP and 81V9 GFP-HFBI).

To validate the observation that both fusion tags appear to increase accumulation levels of the recombinant protein in transgenic plants, statistical analyses of the primary transformants were performed. A normality test was first applied to the recombinant protein quantification data gathered from all of the independent transformants generated per construct. As expected for small populations of first-generation transgenic plants, the assumption of normality was not met [41]. Therefore, a Kruskal-Wallis test (equivalent to parametric analysis of variance, ANOVA) was performed to evaluate if there were statistical differences between the four groups of transgenic lines. The test results demonstrated that there were significant differences between the median values of the four transgenic groups (P < 0.001). Consequently, a Wilcoxon-Mann–Whitney test was applied to compare the four transgenic groups (Figure 3). Results demonstrated that the ELP and HFBI tags have a significant positive effect on the accumulation levels of GFP in stable transgenic plants when compared to GFP with no fusion tag, that HFBI improves GFP accumulation levels significantly more than ELP, and that there is no significant difference between cultivars I64 and 81V9 expressing GFP-HFBI (Figure 3).

### Protein body formation in transgenic *N. tabacum* plants

**Figure 4:**
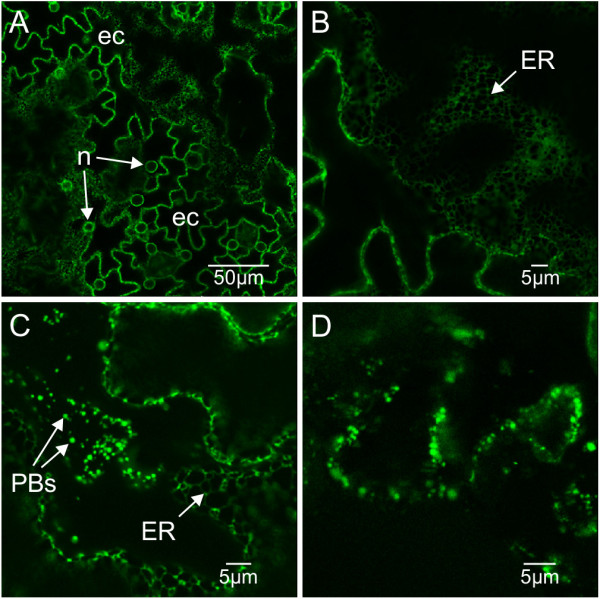
**ER pattern and small PBs in leaf tissues of cv. I64 expressing ER-targeted GFP. A-C**. Leaf cells from cv. I64 plant 18 with GFP accumulation at 0.35% of TSP. Puzzle-shaped epidermal cells (ec) are revealed by GFP fluorescence in the ER. The ER also surrounds the nucleus (n) which appears as circles inside epidermal cells. **B**. Close-up of (**A**) showing the reticulated pattern characteristic of the ER. **C**. Small PBs in another cell of cv. I64 plant 18. **D**. Small PBs in cv. I64 plant 24 (1.2% TSP).

To evaluate the subcellular localization of ER-targeted GFP, GFP-HFBI and GFP-ELP, confocal laser scanning microscopy was performed on several leaves of multiple plants including a young, a medium-sized and an old leaf (Figure 2, Additional file 1: Tables S1, S2, S3 and S4). As untransformed plants showed no evidence of the green fluorescence signal (data not shown), the observed fluorescence in all transgenic lines was attributed to expression of GFP. As expected, a fluorescent reticulated pattern of the ER network was observed with ER-targeted GFP (Figure 4A and 4B). In some cases, GFP-expressing plants displayed small spherical particles ranging between 0.2 – 0.5 μm (Figure 4C and D), similar to those previously reported in transient expression assays in *N. benthamiana* leaves and characterized as PBs [14]. No differences were observed in the fluorescence patterns of young, medium-sized and old leaves in any particular plant.

**Figure 5:**
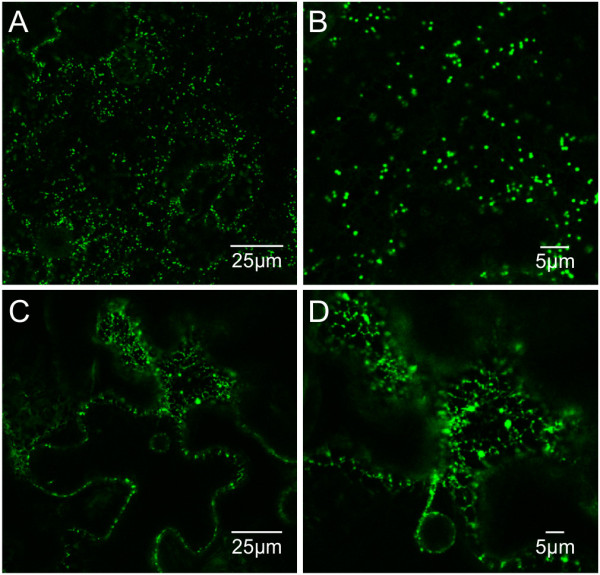
**GFP-ELP induces the formation of PBs in leaves of cv. I64. A-D**. Small PBs form in a T1 progeny of I64 plant 10. **C-D**. Formation of PBs can be seen along the ER network. PBs are heterogeneous in size ranging between 0.5-2 μm.

GFP-ELP transgenic leaf cells displayed PBs in most cells (Figure 5A and 5C). These PBs varied in size, ranging in diameter between 0.5 and 2 μm. In these plants, the simultaneous visualization of PBs and ER was generally difficult due to the brightness of the PBs. The PBs were mostly mobile similar to transiently-induced GFP-ELP PBs in *N. benthamiana* leaves [14]. In some cases, the formation of PBs could be seen along the typical ER network (Figure 5C-D). Also, leaves with different ages displayed similar patterns of PBs.

**Figure 6:**
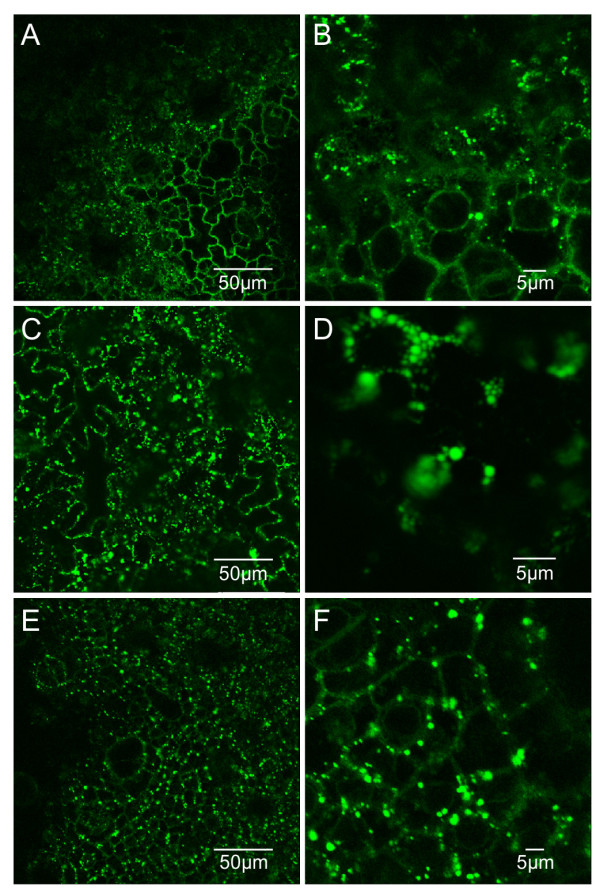
**GFP-HFBI induces the formation of PBs in leaf tissues of transgenic tobacco 81V9 plants. A**. Leaf cells from cultivar 81V9 transgenic plant 11 (0.39% TSP) expressing GFP-HFBI. Formation of PBs is evident. **B**. Close-up of picture A showing PBs ranging between 0.5 - 1.0 μm. **C**. Leaf cells from cultivar 81V9 transgenic plant 19 (1.17% TSP) expressing GFP-HFBI; PBs are more abundant. **D**. Close-up of picture C showing clusters of PBs. **E**. Leaf cells from cultivar I64 transgenic plant 19 (1.68% TSP) expressing GFP-HFBI. Abundant PBs are present. **F**. Close-up of E.

GFP-HFBI transgenic plants of both cultivars I64 and 81V9 showed abundant PBs in leaf cells similar to GFP-ELP transgenic plants (Figure 6A, 6C and 6E). No obvious differences could be detected in distribution patterns of PBs in leaves with different ages or between the two transformed cultivars. Most PBs had a diameter of 0.5 – 1.0 μm (Figure 6B, 6D, 6F), and plants with higher levels of accumulation also had larger PBs (>1.0 μm) more frequently (compare Figure 6B to 6F). The similarity in PB appearance and their correlation with accumulation levels of GFP-HFBI in both tobacco cultivars supports the idea that the HFBI tag behaves similarly in both 81V9 and I64 (Table 2).

**Table 2:** Occurrence and size distribution of PBs in transgenic tobacco with different GFP-HFBI accumulation levels

**Analyzed plants**^**a**^	**GFP-HFBI Accumulation % TSP**	**Protein bodies**	**Protein body size**		
		**Absence**^**b**^	**Presence**	**Small**^**c**^	**Large**^**d**^
I64 GFP-HFBI	0.55%	0	100	97%	3%
I64 GFP-HFBI	1.68%	0	100	37%	63%
81V9 GFP-HFBI	0.77%	0	100	87%	13%
81V9 GFP-HFBI .	1.17%	0	100	48%	52%

^**a**^, In total, five leaves per plant were sampled. Two leaf discs per leaf were collected and analyzed by laser scanning confocal microscopy. Ten cells were analyzed per leaf disc, for a total of 100 cells to avoid any bias.

^**b**^, Cells displaying a bright fluorescent ER reticulated pattern.

^**c**^, PB diameter <1.0 μm.

^**d**^, PB diameter >1.0 μm.

**Figure 7:**
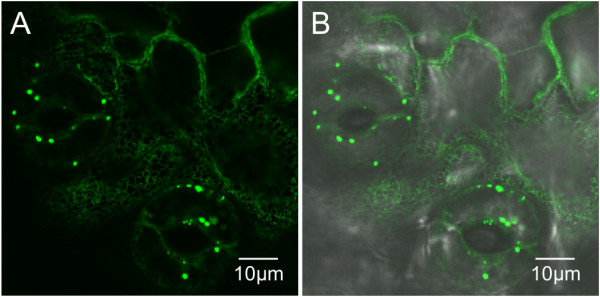
**Guard cells from leaf tissue of a transgenic tobacco expressing GFP-HFBI contain PBs.** PBs in guard cells from cv. 81V9 transgenic plant expressing GFP-HFBI. **A**. GFP signal from PBs in guard cells and ER pattern in surrounding epidermal cells. **B**. Overlay of the GFP green channel with the transmission light channel confirming the localization of the PBs to the guard cells.

An interesting observation is the presence of PBs in guard cells of stomata in most plants that formed PBs regardless of the cultivar and the construct. These cells had often more PBs than the surrounding epidermal cells (Figure 7).

### A threshold value of recombinant protein accumulation is required for protein body formation in transgenic tobacco plants

A comparison of the confocal laser scanning microscopy results and the obtained accumulation levels of different transgenic lines was performed. This analysis revealed a relationship between PB formation and recombinant protein accumulation. Essentially, higher accumulation levels of GFP were associated with the presence of PBs. Based on these findings, all transgenic lines were divided into two different classes according to accumulation levels and presence or absence of PBs (Table 3). The first class represents plants with low accumulation levels, ranging between 0.01% and 0.2% of TSP showing no PBs. The second class represents plants with accumulation levels of 0.2% of TSP and above. In this class, PBs form in almost every cell. Moreover, PB frequency and size increase with accumulation levels (Table 2). According to our results, we hypothesize that a threshold of GFP accumulation around 0.2% of TSP is needed for the formation of PBs in leaves of transgenic tobacco, regardless of the construct and cultivar used. This hypothesis is supported by the fact that in 93.3% of the analyzed plants this model is fulfilled (Table 3).

**Table 3:** Correlation between GFP accumulation levels and PB presence in plants expressing GFP, GFP-ELP and GFP-HFBI

**Accumulation level of GFP**^**a**^	**PBs**	
	**Absence**	**Presence**
Less than 0.2% TSP	20	0
More than 0.2% TSP	4	36

^a^, In total, 60 transgenic lines were analyzed by laser scanning confocal microscopy to determine presence or absences of PBs. Three leaf samples per plant were analyzed including one young leaf, one medium-sized leaf and one old leaf.

### Evidence of post-transcriptional gene silencing in low expressing transgenic plants

Among the stable transgenic lines generated in this study, some lines yielded low accumulation levels. The presence of plants with such low accumulation levels is frequently explained by positional effects of transgene insertion in the genome [42] or silencing mechanisms of the plant [43]. The latter is usually attributed to post-transcriptional gene silencing (PTGS), a ribonucleic acid (RNA) based silencing mechanism that can be activated by plant pathogens, transposons and transgenes [44,45].

**Figure 8:**
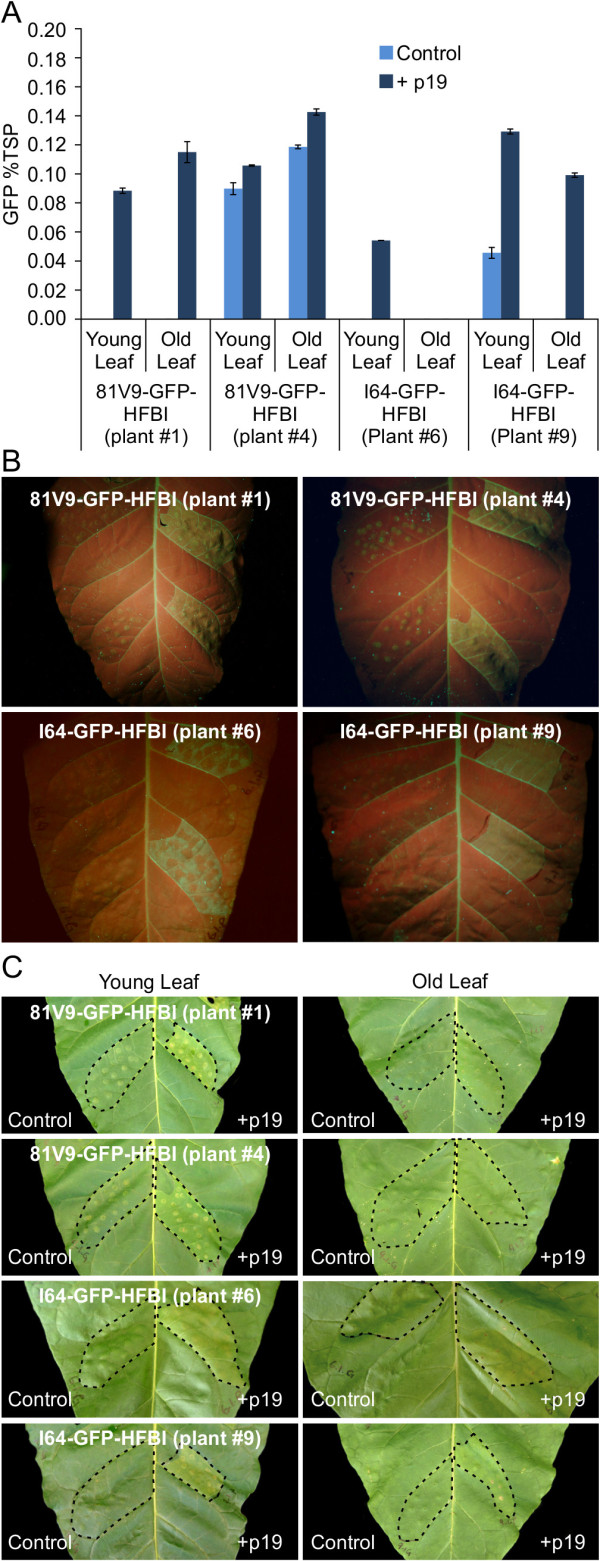
**Effect of p19 suppressor of gene silencing on transgenic tobacco lines with low accumulation levels. A**. Quantification of GFP expression in infiltrated tissue of young and old leaves. Light blue represents the mock-infiltrated area with Gamborg’s solution (control). Dark blue represents the p19 infiltrated tissue. Error bars represent technical replicates of GFP quantitation in agro-infiltrated sectors. Absence of bars indicates no GFP detection **B**. UV visualization of p19-infiltrated and mock-infiltrated tissues at 2 dpi in young leaves. The p19-infiltrated sectors (right) fluoresce green compared to the mock-infiltrated sectors (left). **C**. Daylight visualization of p19-infiltrated and mock-infiltrated young and old leaves at 5 dpi; slight chlorosis is apparent in young leaves only. Infiltrated areas are delineated with dotted black lines.

To evaluate if the low accumulation levels of some of our transgenic plants was due to an active PTGS mechanism, four GFP-HFBI T_1_ progeny plants of T_0_ lines with low accumulation levels were transiently transformed with an *Agrobacterium* strain carrying the p19 gene from *Cymbidium* ringspot virus (CymRSV) [46]. To eliminate any effects due to leaf age and developmental stage, two leaves (young and old) on each plant were agro-infiltrated. Starting two days post infiltration (2 dpi), a significant increase in GFP accumulation was observed in the p19 infiltrated tissue compared with the control (Gamborg’s solution). The GFP signal from the infiltrated tissue was bright enough to be easily visualized with a UV light. Quantitative analysis of the p19-infiltrated and control tissue confirmed the positive effect of p19 on increasing GFP expression levels regardless of cultivar type (81V9 vs. I64) or leaf age (young or old leaves) (Figure 8A-B). These results indicate that low accumulation levels of a recombinant protein in stable transgenic plants may be at least partly attributed to PTGS. PB formation was investigated in p19 infiltrated leaf tissue at 2 and 5 dpi. No PBs were observed in any of the infiltrated lines. Given that GFP accumulation levels for all infiltrated and control tissue were below 0.2% of TSP (Figure 8A), these results are consistent with the idea that PBs appear at accumulation levels higher than 0.2% of TSP.

One of the major advantages of transgenic tobacco for production of recombinant proteins is the high biomass yield. It has been previously shown that p19 from tomato bushy stunt virus (TBSV) induces a hypersensitive response in tobacco cv. I64 infiltrated tissue which not only reduces the biomass yield of tobacco plants but also negatively affects the accumulation levels of co-expressed proteins starting at 3 dpi [47,48]. We monitored infiltrated 81V9 and I64 leaves from 2 to 5 dpi, and did not observe any discoloration or other signs of necrosis before 5 dpi. On day 5 post infiltration, some slight discoloration was detected mostly in the younger leaves (Figure 8C). Notably, p19 infiltrated tissue yielded higher amounts of GFP compared to mock-infiltrated tissue, contrary to the results obtained with p19 from TBSV [48].

## Discussion

### ELP and HFBI fusions improve accumulation levels of GFP in transgenic tobacco plants

Fusion partners for the expression of recombinant proteins in plants have been used to solve two major problems: low accumulation levels and lack of efficient purification methods for plant made proteins [8]. The ELP and HFBI fusion tags have been tested in transient expression experiments and shown to increase recombinant protein accumulation levels [13,14,30]. ELP was also shown to increase the accumulation of recombinant proteins in stable transgenic plants [49,50], while HFBI has not been tested in a stable plant system and neither were investigated for the appearance of PBs in stable transgenic plants.

Analysis of the various transgenic tobacco plants produced in this study demonstrated that ELP and HFBI tags can be efficiently used in transgenic plants. Compared with unfused GFP, the use of both tags at least doubled the amount of GFP (Figure 3). To our knowledge, this is the first report showing that the HFBI tag is functional in transgenic plants, where it positively impacts the accumulation of the protein of interest. Additionally, no differences were found between cultivars I64 and 81V9 transformed with GFP-HFBI (Figure 3). This finding opens the possibility of using cultivars with different properties that can be beneficial for the expression of a particular protein. Cultivar I64 produces high biomass and normal alkaloid levels, while 81V9 cultivar is a low alkaloid cultivar with slightly lower biomass [9], a property that can be exploited for oral administration of therapeutic biologicals such as vaccines for humans and animals. Expensive purification processes can thus be avoided, and lower product costs can be achieved [51,52].

This is also the first study to directly compare ELP and HFBI tags. We found that ELP increased the accumulation levels of GFP (Figure 2) to a lesser extent than HFBI. This result is consistent with published transient expression data of the same constructs [14,30]. However, a similar comparison with other recombinant proteins should be conducted before definitive conclusions can be drawn since the conformation of individual proteins could be affected by the fusion tags, especially proteins that require complex folding and assembly.

### PB formation is induced by high accumulation of recombinant proteins

The ER is the first gateway of the protein secretory pathway. In cereal seeds, the endomembrane system is able to generate multiple ER-derived compartments or PBs, primarily used to store reserve compounds, such as storage proteins, lipids, carbohydrates and minerals. As such, protein bodies provide a suitable environment for folding, assembling, and long-term storage of massive amounts of proteins [53,54]. In this study, we show that PBs form in leaves of transgenic plants expressing ER-targeted GFP, GFP-ELP and GFP-HFBI at levels higher than 0.2% of TSP. To our knowledge, this is the first report that demonstrates that there is a threshold value needed for the formation of PBs in tobacco leaves, and that PBs are not exclusively induced by the presence of the fusion tags. Indeed, despite an overall lower accumulation of unfused GFP, PBs were found in four of the six transgenic plants that accumulated more than 0.2% of TSP and were examined by confocal microscopy. This finding suggests that the ELP and HFBI tags are not essential for the formation of PBs, although they help to increase the accumulation levels and therefore enhance PBs formation.

### Evidence of post-transcriptional gene silencing in plants with low levels of GFP accumulation

Plant pathogenic viruses have evolved strong PTGS suppressors that can act at different levels of the silencing pathway. One of the best characterized suppressors, expressed by members of the *Tombusvirus* family, is the p19 protein. The p19 suppressor has been successfully used in transient expression assays for increasing the yield of several recombinant proteins [55-58]. The observed variation in accumulation levels of recombinant proteins in different stable transgenic plants can be induced by the activation of plant PTGS mechanism, a silencing system that can be activated by transgenes if their expression levels are high [55,59,60].

Results from agro-infiltration of CymRSV p19 in four of the low expressing GFP-HFBI tobacco plants suggest that some of these plants may have been silenced. This was shown by the observed increase in accumulation of the recombinant protein after infiltration with p19. CymRSV p19 infiltration of tobacco cultivars I64 and 81V9 did not cause necrosis in the infiltrated areas, differing from a recent report where p19 from TBSV- induced necrosis in several tobacco cultivars including cv. I64 starting at 3 dpi [48]. Other than different origins of the p19 proteins used in these two studies, the age at which tobacco plants were infected with the p19 varied. In our case, the infiltrated tobacco cultivars were 12–14 weeks old while Garabagi *et al.*[48] used 6–8 week-old plants. In their experiment Garabagi *et al.*[48] observed complete necrosis by 5 dpi in cv. I64, while we observed slight yellowing of infiltrated leaf sectors in young leaves only and no effect on older leaves of 12–14 week-old plants. The difference in results could be due to different p19 genes which are identical at 68% of amino acid positions [47] but also to the age of the plant as well as the developmental stage of the leaves.

## Conclusions

In this study, we have shown that ELP and HFBI fusion tags expressed in two different cultivars of transgenic tobacco, help to increase the accumulation level of recombinant GFP, and induce formation of PBs. We have also shown that a threshold concentration (0.2% of TSP) of GFP was required for PBs to form, regardless of the fusion partner’s presence or absence. Additionally, we have shown that PTGS can play a role in expression and accumulation of recombinant proteins in transgenic plants.

## Methods

### Generation of tobacco stable transgenic plants

Previously published constructs were used for tobacco stable transformation [14,30] (Figure 1). Tobacco cultivars I64 and 81V9 were transformed using *Agrobacterium* according to Horsch *et al.*[61]. Tobacco plants were grown in a greenhouse at 24°C, 16 hour light and 22°C, 8 hour dark for 12–14 weeks. Plants were fertilized with the classic water soluble fertilizer (N : P : K = 20 : 20 : 20) weekly at 4 g/L (Plant Products, Brampton, ON, Canada).

### Transient expression in tobacco stable transgenic plants

Leaves of tobacco cultivars 81V9 and I64 expressing GFP-HFBI were agro-infiltrated [62] using a needle-less syringe with an *Agrobacterium* strain containing p19 from CymRSV [46]. Briefly, the *A. tumefaciens* culture was grown to an optical density at 600 nm (OD_600_) of 0.5-0.8. The culture was centrifuged at 1000 g for 30 minutes and the pellet was resuspended in agro-infiltration solution (3.2 g/L Gamborg’s B5 plus vitamins, 20 g/L sucrose, 10 mM MES pH 5.6, 200 μM 4′-Hydroxy-3′,5′-dimethoxyacetophenone) to a final OD_600_ of 0.3 and then incubated at room temperature with gentle agitation for 1 hour. A young leaf (the third leaf from the top of the plant), a medium-sized leaf and an old leaf (the 8th leaf from the top) from different stable transgenic plants were infiltrated with the bacterial suspension. Leaf tissue samples were collected 5 days post infiltration.

### Tissue sample collection and protein extraction

Leaf tissue samples were collected from each stable transgenic plant when the plant had 8 fully expanded leaves. In total, 8 leaf discs, 5 mm in diameter, were collected per plant, each from a different leaf and froze instantly in liquid nitrogen. Frozen leaf discs were homogenized using a Tissue Lyser (Qiagen), extracted in 600 μl of plant protein extraction buffer containing phosphate-buffered saline (PBS: 8 g/L NaCl, 1.16 g/L Na_2_HPO_4_, 0.2 g/L KH_2_PO_4_, 0.2 g/L KCl, pH 7.4), 1 mM EDTA, 1 mM phenylmethanesulfonylfluoride (PMSF), 1 μg/ml leupeptin 0.1% Tween-20, 100 mM sodium L-ascorbate, and centrifuged twice at 14,000 g for 5 minutes at 4°C. The extracted proteins in the supernatant were kept on ice, TSP was determined by a Bradford assay using bovine serum albumin (BSA) as a standard (Bio-Rad) [63].

### Quantification of GFP levels

GFP quantification of the recombinant proteins was performed either using the immunodot blot technique or fluorometer. For immunodot blot quantitation, serial dilutions of each sample were prepared and three technical replicates of each dilution were spotted onto a nitrocellulose membrane (Bio-Rad). A dilution series of GFP-HFBI, purified by ATPS, was used as a standard on every blot to quantify the amount of GFP. Membranes were blocked overnight at 4°C in blocking solution containing tris-buffered saline (TBS-T 24.2 g/L Tris base, 175.3 g/L NaCl, pH 7.5, 0.1% Tween-20) and 3% (w/v) powdered skim milk. Membranes were incubated for 1 hour at room temperature with mouse monoclonal anti GFP primary antibody (Clontech, Living Colors® A.v. monoclonal antibody (JL-8), Cat. No. 632381) diluted 1:5000 in blocking solution. Membranes were washed three times for 15 minutes with TBS-T and incubated for an hour at room temperature with a horseradish peroxidase (HRP)-conjugated goat-anti mouse IgG secondary antibody (Bio-Rad, Cat. No. 170–6515) diluted in 1:3000 in blocking solution. Membranes were visualized using the enhanced chemiluminescence (ECL) detection system (GE Healthcare, Mississauga, ON, Canada) according to the manufacturers protocol, and scanned using a ChemidocXRS (Bio-Rad). Scanned membranes were analyzed using TotalLab TL 100 software (Nonlinear Dynamics, Durham, USA). All sample dilutions were compared to a GFP-HFBI standard that was produced in the lab. For this purpose, *Agrobacterium*-mediated transient expression was used to produce GFP-HFBI recombinant protein in *N. benthamiana* plants. The recombinant protein was purified from infiltrated leaf tissue using ATPS according to Joensuu *et al.*[30] and quantified using SDS-PAGE. GFP-HFBI at 4, 8, 12, 16, 20 and 24 ng/μl were prepared and used to create a standard curve for extrapolating concentrations of GFP in transgenic plants.

GFP was quantified by fluorometry in leaf extracts by measuring fluorescent intensity with a Synergy™ 2 microplate reader (BioTek, VT, USA). Briefly, 200 μl of serially diluted samples in PBS were added to 96 well black opaque flat bottom polystyrene TC-treated microplates (Corning, MA, USA). Fluorescence was detected using excitation and emission at 485 and 516 nm, respectively. To account for background fluorescence of the plant tissue, extract from mock-infiltrated tissue with Gamborg’s solution was used to normalize the data. All sample dilutions were compared to a GFP-HFBI standard. Several concentrations of purified GFP were used (ranging between 125 to 2000 ng/ml) to draw the standard curve. The mean of three technical replicates of each standard point and diluted sample was used as the credible value.

### Statistical analysis

Minitab 15 statistical package (Minitab Ltd., Coventry, UK) was used to perform the statistical analysis. Kolmogorov-Smirnov normality test (Lilliefor’s test) was first applied to the quantification data gathered from the 24 independent transformants that were regenerated per transformed construct (96 in total). The Kruskal-Wallis non-parametric test was used to assess if there were statistical differences between the median values of the four different transgenic groups. Post-hoc comparisons between groups of transgenic lines were performed using the Wilcoxon-Mann–Whitney test.

### Tissue sampling of stable transgenic plants and confocal microscopy analysis

Tissue samples were collected from fully expanded and developed leaves. In total, 3 leaf discs, 4 mm in diameter, were collected per plant, each from a different size leaf (one from a young leaf (third leaf from the top of the plant), one from a medium-size leaf (5th leaf from the top) and one from an old leaf (8th leaf from the top)). Samples were immediately imaged with a Leica TCS SP2 confocal laser scanning inverted microscope (Leica Microsystems, Wetzlar, Germany) equipped with a 63X water immersion objective. To visualize GFP fluorescence, excitation with a 488 nm argon laser was used and the emission was detected at 500–530 nm. Collected images were analyzed using the Leica Application Suite for Advanced Fluorescence (LAS AF, V2.3.5) (Leica Microsystem, Germany).

## Abbreviations

ATPS: Aqueous two-phase system; CymRSV: Cymbidium ringspot tombusvirus; ELP: Elastin-like polypeptide; GFP: Green fluorescent protein; HFBI: Hydrophobin I; ITC: Inverse transition cycling; PB: Protein body; PTGS: Post-transcriptional gene silencing; TSP: Total soluble protein.

## Competing interests

The authors declare that they have no competing interests.

## Authors’ contributions

RM conceived the study. SPG and RS designed and performed the experiments, and analyzed the data; RM and SK participated in designing the study and supervised the work. All authors read and approved the final manuscript.

## Supplementary Material

Additional file 1: Table S1 GFP accumulation levels in transgenic tobacco cv. I64. Table S2. GFP-ELP accumulation levels in transgenic *N. tabacum* cv. I64. Table S3. GFP-HFBI accumulation levels in transgenic *N. tabacum* cv. I64. Table S4. GFP-HFBI accumulation levels in transgenic *N. tabacum* cv. 81V9. Click here for file
