# Supplementary material for: Protein body formation in stable transgenic tobacco expressing elastin-like polypeptide and hydrophobin fusion proteins
Source: BMC Biotechnol. 2013 May 10;13:40. doi: 10.1186/1472-6750-13-40 (PMC3659085; doi:10.1186/1472-6750-13-40)
Supplement: Additional file 1: Table S1 — GFP accumulation levels in transgenic tobacco cv. I64. Table S2. GFP-ELP accumulation levels in transgenic N. tabacum cv. I64. Table S3. GFP-HFBI accumulation levels in transgenic N. tabacum cv. I64. Table S4. GFP-HFBI accumulation levels in transgenic N. tabacum cv. 81V9. [file 1472-6750-13-40-S1.docx]

Table 1. GFP accumulation levels in transgenic tobacco cv. I64.

| **Transgenic line** | **Recombinant protein** [%**TSP**]**^a^** | **PBs^b^** |
| --- | --- | --- |
| 1 | 0.014 | A |
| 2 | 0.018 | A |
| 3 | 0.022 | A |
| 4 | 0.031 | ND |
| 5 | 0.031 | A |
| 6 | 0.032 | A |
| 7 | 0.041 | ND |
| 8 | 0.049 | A |
| 9 | 0.054 | A |
| 10 | 0.060 | A |
| 11 | 0.074 | ND |
| 12 | 0.090 | ND |
| 13 | 0.106 | ND |
| 14 | 0.112 | A |
| 15 | 0.227 | A |
| 16 | 0.237 | P |
| 17 | 0.321 | P |
| 18 | 0.354 | P |
| 19 | 0.457 | ND |
| 20 | 0.460 | A |
| 21 | 0.631 | ND |
| 22 | 0.810 | ND |
| 23 | 1.142 | ND |
| 24 | 1.201 | P |
|  |  |  |

^a^, Twenty four independent transformants were evaluated. The quantity of recombinant protein present in each sample was calculated as the mean of three technical replicates in immunodot blotting.

^b^, Presence (P) or absence (A) of PBs in transgenic lines. ND, not determined.

**Table 2. GFP-ELP accumulation levels in transgenic *N. tabacum* cv. I64.**

| **Transgenic line** | **Recombinant protein** [% **per TSP**]**^a^** | **PBs^b^** | |  |
| --- | --- | --- | --- | --- |
| 1 | 0.020 | A | |  |
| 2 | 0.031 | A | |  |
| 3 | 0.043 | A | |  |
| 4 | 0.074 | A | |  |
| 5 | 0.085 | A | |  |
| 6 | 0.094 | ND | |  |
| 7 | 0.106 | ND | |  |
| 8 | 0.108 | A | |  |
| 9 | 0.126 | A | |  |
| 10 | 0.193 | ND | |  |
| 11 | 0.210 | P | |  |
| 12 | 0.224 | P | |  |
| 13 | 0.236 | P | |  |
| 14 | 0.303 | P | |  |
| 15 | 0.325 | P | |  |
| 16 | 0.404 | P | |  |
| 17 | 0.599 | P | |  |
| 18 | 0.715 | | ND | |
| 19 | 0.813 | | P | |
| 20 | 0.877 | | ND | |
| 21 | 1.085 | | A | |
| 22 | 1.449 | | ND | |
| 23 | 1.722 | | ND | |
| 24 | 2.100 | | P | |
|  |  | |  | |

^a^, Twenty four independent transformants were evaluated. The quantity of recombinant protein present in each sample was calculated as the mean of three technical replicates in immunodot blotting.

^b^, Presence (P) or absence (A) of PBs in transgenic lines. ND, not determined.

Table 3. GFP-HFBI accumulation levels in transgenic *N. tabacum* cv. I64.

| **Transgenic line** | **Recombinant protein** [% **per TSP**]**^a^** | **PBs^b^** |
| --- | --- | --- |
| 1 | 0.018 | A |
| 2 | 0.100 | A |
| 3 | 0.122 | A |
| 4 | 0.149 | ND |
| 5 | 0.169 | A |
| 6 | 0.172 | ND |
| 7 | 0.219 | ND |
| 8 | 0.232 | ND |
| 9 | 0.255 | ND |
| 10 | 0.294 | ND |
| 11 | 0.349 | P |
| 12 | 0.451 | ND |
| 13 | 0.510 | P |
| 14 | 0.553 | P |
| 15 | 0.779 | P |
| 16 | 0.782 | P |
| 17 | 0.999 | ND |
| 18 | 1.514 | P |
| 19 | 1.684 | P |
| 20 | 1.754 | P |
| 21 | 1.935 | ND |
| 22 | 2.141 | P |
| 23 | 2.271 | ND |
| 24 | 2.521 | P |
|  |  |  |

^a^, Twenty four independent transformants were evaluated. The quantity of recombinant protein present in each sample was calculated as the mean of three technical replicates in immunodot blotting.

**^b^**, Presence (P) or absence (A) of PBs in transgenic lines. ND, not determined.

Table 4. GFP-HFBI accumulation levels in transgenic *N. tabacum* cv. 81V9.

| **Transgenic line** | **Recombinant protein** [% **per TSP**]**^a^** | **PBs^b^** |
| --- | --- | --- |
| 1 | 0.102 | ND |
| 2 | 0.136 | ND |
| 3 | 0.179 | ND |
| 4 | 0.196 | ND |
| 5 | 0.218 | ND |
| 6 | 0.225 | P |
| 7 | 0.263 | P |
| 8 | 0.294 | P |
| 9 | 0.351 | P |
| 10 | 0.357 | P |
| 11 | 0.397 | P |
| 12 | 0.404 | P |
| 13 | 0.776 | P |
| 14 | 0.808 | ND |
| 15 | 0.846 | P |
| 16 | 1.0 | ND |
| 17 | 1.051 | P |
| 18 | 1.098 | ND |
| 19 | 1.171 | P |
| 20 | 1.212 | A |
| 21 | 1.371 | ND |
| 22 | 2.066 | P |
| 23 | 2.507 | ND |
| 24 | 5.226 | P |
|  |  |  |

^a^, Twenty four independent transformants were evaluated. The quantity of recombinant protein present in each sample was calculated as the mean of three technical replicates in immunodot blotting.

^b^, Presence (P) or absence (A) of PBs in stable transgenic lines determined by confocal microscopy analysis. ND, not determined.
